# Magnetic critical behavior of the van der Waals Fe_5_GeTe_2_ crystal with near room temperature ferromagnetism

**DOI:** 10.1038/s41598-020-72203-3

**Published:** 2020-09-18

**Authors:** Zhengxian Li, Wei Xia, Hao Su, Zhenhai Yu, Yunpeng Fu, Leiming Chen, Xia Wang, Na Yu, Zhiqiang Zou, Yanfeng Guo

**Affiliations:** 1 School of Physical Science and Technology, ShanghaiTech University, Shanghai, 201210 China; 2 Shanghai Institute of Optics and Fine Mechanics, Chinese Academy of Sciences, Shanghai, 201800 China; 3 University of Chinese Academy of Sciences, Beijing, 100049 China; 4 School of Materials Science and Engineering, Henan Key Laboratory of Aeronautic Materials and Application Technology, Zhengzhou University of Aeronautics, Zhengzhou, 450046 Henan China; 5 Analytical Instrumentation Center, School of Physical Science and Technology, ShanghaiTech University, Shanghai, 201210 China

**Keywords:** Physics, Condensed-matter physics, Ferromagnetism

## Abstract

The van der Waals ferromagnet Fe_5_GeTe_2_ has a Curie temperature *T*_C_ of about 270 K, which is tunable through controlling the Fe deficiency content and can even reach above room temperature. To achieve insights into its ferromagnetic exchange that gives the high *T*_C_, the critical behavior has been investigated by measuring the magnetization in Fe_5_GeTe_2_ crystal around the ferromagnetic ordering temperature. The analysis of the measured magnetization by using various techniques harmonically reached to a set of reliable critical exponents with *T*_C_ = 273.7 K, *β* = 0.3457 ± 0.001, *γ* = 1.40617 ± 0.003, and *δ* = 5.021 ± 0.001. By comparing these critical exponents with those predicted by various models, it seems that the magnetic properties of Fe_5_GeTe_2_ could be interpreted by a three-dimensional magnetic exchange with the exchange distance decaying as *J*(*r*) ≈ *r*^−4.916^, close to that of a three-dimensional Heisenberg model with long-range magnetic coupling.

## Introduction

A prominent virtue of the quasi-two-dimensional (2D) van der Waals (vdW) bonded materials is that they could be exfoliated into multi- or single layer, thus making them useful in various novel heterostructures and devices. Moreover, the vdW materials in the 2D limit exhibit extraordinary physical properties, such as those observed in the intensively studied graphene and transition metal dichalcogenides^1–6^, etc. Known as the Merin-Wagner theorem^7^, intrinsic long-range magnetic order can not appear in the isotropic magnetic 2D limit because the strong thermal fluctuations in such case prohibit the spontaneous symmetry breaking and hence the long-range magnetic ordering. Nevertheless, a small anisotropy is sufficient to open up a sizable gap in the magnon spectra and consequently stabilizes the magnetic order against finite temperature. This picture has been realized by the observation of long-range ferromagnetic (FM) order in mono- or few-layer CrI_3_^8^, Cr_2_Ge_2_Te_6_^9^, Cr_2_Si_2_Te_6_^10^, VSe_2_^11^, and MnSe_2_^12^, etc. The vdW magnets in the 2D limit host rich magneto-electrical, magneto-optical, or spin–lattice coupling effects that are capable of producing intriguing properties which are scarcely observed in bulk. Very recently, current-induced magnetic switch was observed in the few-layer Fe_3_GeTe_2_^13^, demonstrating the vdW magnets a versatile platform for nanoelctronics. Moreover, heterostructures constructed by using vdW magnets have profound valleytronics and spintronics device applications^14,15^. For example, the tunneling magnetoresistance (MR) in spin-filter magnetic vdW CrI_3_ heterostructures even approaches 1.9 × 10^4^%, remarkably superior to that constructed by using conventional magnetic thin films^16^. The easy exfoliation, weak interlayer coupling, and tunability of magnetic properties make the vdW magnets a model family of materials for exploring exotic phenomena and finding novel applications.

In the handful FM vdW magnets, the physical properties in the 2D limit differ from each other due to rather complex magnetic interactions. The semiconducting monolayer CrI_3_ is an Ising ferromagnet with very low Curie temperature (*T*_C_) of about 45 K due to the weak superexchange interaction along the Cr-I-Cr pathway^8,17^. The similar weak FM superexchange in the Heisenberg magnet bilayer Cr_2_Ge_2_Te_3_ also results in a low *T*_C_ of ~ 30 K, and FM order is even not present in the monolayer^9^. As a contrast, the FM exchange with an itinerant character mediated by carriers in metallic Fe_3_GeTe_2_ monolayer is much stronger than the superexchange in CrI_3_ and Cr_2_Ge_2_Te_6_, thus yielding a remarkably higher *T*_C_ of about ~ 130 K, which can be raised even above room temperature by using the ionic gating technique^18,19^.

The tremendous efforts in perusing high *T*_C_ magnets more recently led to the discovery of a *T*_C_ of ~ 130–230 K in the bulk quasi-2D vdW Fe_3-x_GeTe_2_, which can even be enhanced up to room temperature^18^. Interestingly, similar as Fe_3-*x*_GeTe_2_, bulk Fe_5-*x*_GeTe_2_ shows a tunable *T*_C_ ranging from ~ 270 to ~ 363 K by controlling the Fe deficiency content *x* or by substituting Co for Fe, suggesting the detrimental role of Fe in the magnetic exchange^20,21,56^. A reversible magnetoelastic coupled first-order transition near 100 K was detected by neutron powder diffraction^20^. Considering the exotic physical properties in exfoliated Fe_3_GeTe_2_ nanoflakes and its heterostructures, such as the extremely large anomalous Hall effect^22^, planar topological Hall effect^23^, Kondo lattice physics^24^, anisotropic magnetostriction effect^25^, spin filtered tunneling effect^16^, magnetic skyrmions^26^, etc., Fe_5_GeTe_2_ would also be expected to provide extraordinary opportunities to explore intriguing physical properties. To well understand the physical properties of Fe_5_GeTe_2_, the magnetic exchange model should be established first. However, the direct measurements on the magnetic structure are absent yet. Alternatively, study on the magnetic critical behavior and analysis of the critical exponents in vicinity of the paramagnetic (PM) to FM transition region could yield valuable insights into the magnetic exchange and properties. For example, the method has established the magnetic exchange models for CrI_3_^27^, VI_3_^28^, Fe_3_GeTe_2_^29,30^, Co_2_TiSe^31^, and Fe_0.26_TaS_2_^32^, etc. In this work, we have reported the investigation on the critical behavior of Fe_5_GeTe_2_, which finds that the obtained set of critical exponents are close to those calculated from the renormalization group approach for a long-range 3D Heisenberg model with the magnetic exchange distance decaying as *J*(*r*) ≈ *r*^−4.916^.

## Result and discussion

**Figure 1:**
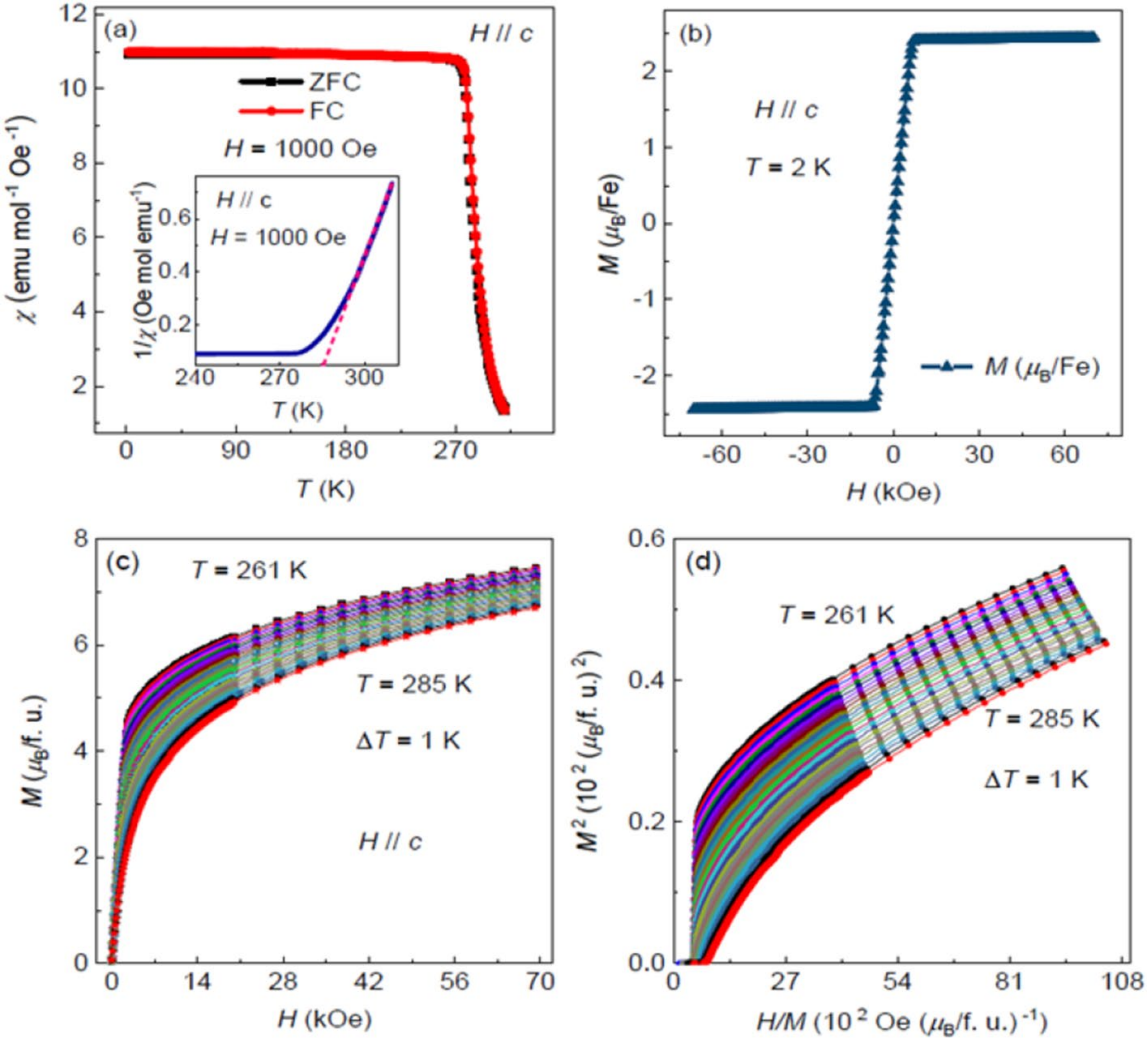
(**a**) Temperature dependence of magnetization *M*(*T*) for Fe_5_GeTe_2_ under *H* = 1 kOe. The inset shows the inverse susceptibility plotted against temperature and the straight dotted line is Curie–Weiss law fitting. (**b**) Isothermal magnetization *M*(*H*) measured at 2 K. (**c**) Typical initial magnetization *M*(*H*) curves measured from 261 to 285 K with an interval of 1 K. (**d**) Arrott plots in the form of *M*^2^ vs. *H*/*M* (mean field model) around *T*_C_.

Chracterizations on the crystal structure, quality and compositions are presented in the supplementary materials (SI). Figure 1a depicts the temperature dependence of magnetization *M*(*T*) for Fe_5_GeTe_2_ measured with zero-field-cooling (ZFC) and field-cooling (FC) mode under the applied magnetic field *H* = 1 kOe along the *ab*-plane of the crystal. The magnetization displays an abrupt PM to FM transition at ~ 270 K and no clear separation between the ZFC and FC curves. The inset of Fig. 1a is the inverse temperature dependent magnetic susceptibility *χ*^-1^(*T*) with the dotted straight line representing the Curie–Weiss law fitting. It shows a deviation of ${\chi }^{-1}(T)$ from the straight line near 295 K which is much higher than *T*_C_. The obtained Weiss temperature is 283 K, which is also higher than *T*_C_, indicating a strong FM interaction. The effective moment as *μ*_eff_ = 6.659 *μ*_B_/Fe is also obtained. Considering the varied effective magnetic moment of Fe^2+^ with the values raging from 4.90 to 6.70 *μ*_B_ in various materials including sphalerite and monoclinic pyroxenes obtained from magnetic susceptibility analysis^33^ and the Fe deficiency in our crystals, the value we obtained from the Curie–Weiss law fitting is reasonable. The FM ground state can also be demonstrated by the isothermal magnetization *M*(*H*) shown in Fig. 1b measured at 2 K. The low coercive field indicates a soft ferromagnetism in Fe_5_GeTe_2_, which is similar as that of Fe_3_GeTe_2_^29,30^. The saturation magnetic moment along the *c*-axis is about 2.4 *μ*_B_/Fe, likely unveiling the magnetic anisotropy at low temperature. The initial isothermal magnetizations in the temperature range of 261–285 K measured with *H*//*c*-axis were shown in Fig. 1c and the Arrott plot^34^, that is, *M*^2^ vs. *H*//*M*, is shown in Fig. 1d. The positive slope of all *M*^2^ vs. *H*/*M* curves, according to the Banerjee’s criterion^35^, indicates that the PM to FM transition has a second-order in nature. The Arrott plot was initially tried to for the analysis of the measured magnetizations, so the mean Landau mean-field theory with the critical exponents *β* = 0.5 and *γ* = 1.0 is involved. If it works, the *M*^2^ vs. *H*//*M* curves should be straight and parallel to each other in the high magnetic field region, and additionally, the isothermal magnetization at *T*_C_ should pass through the origin. However, seen in Fig. 1d *M*^2^ vs. *H*//*M* curves are clearly nonlinear with a downward curvature, suggesting that the fit does not work for Fe_5_GeTe_2_. The failure of the Arrott plot within the framework of Landau mean-field theory lies in that the itinerant ferromagnetism in Fe_5_GeTe_2_ should have significant electronic correlations and spin fluctuations, which however are neglected in the Landau mean-field theory.

**Figure 2:**
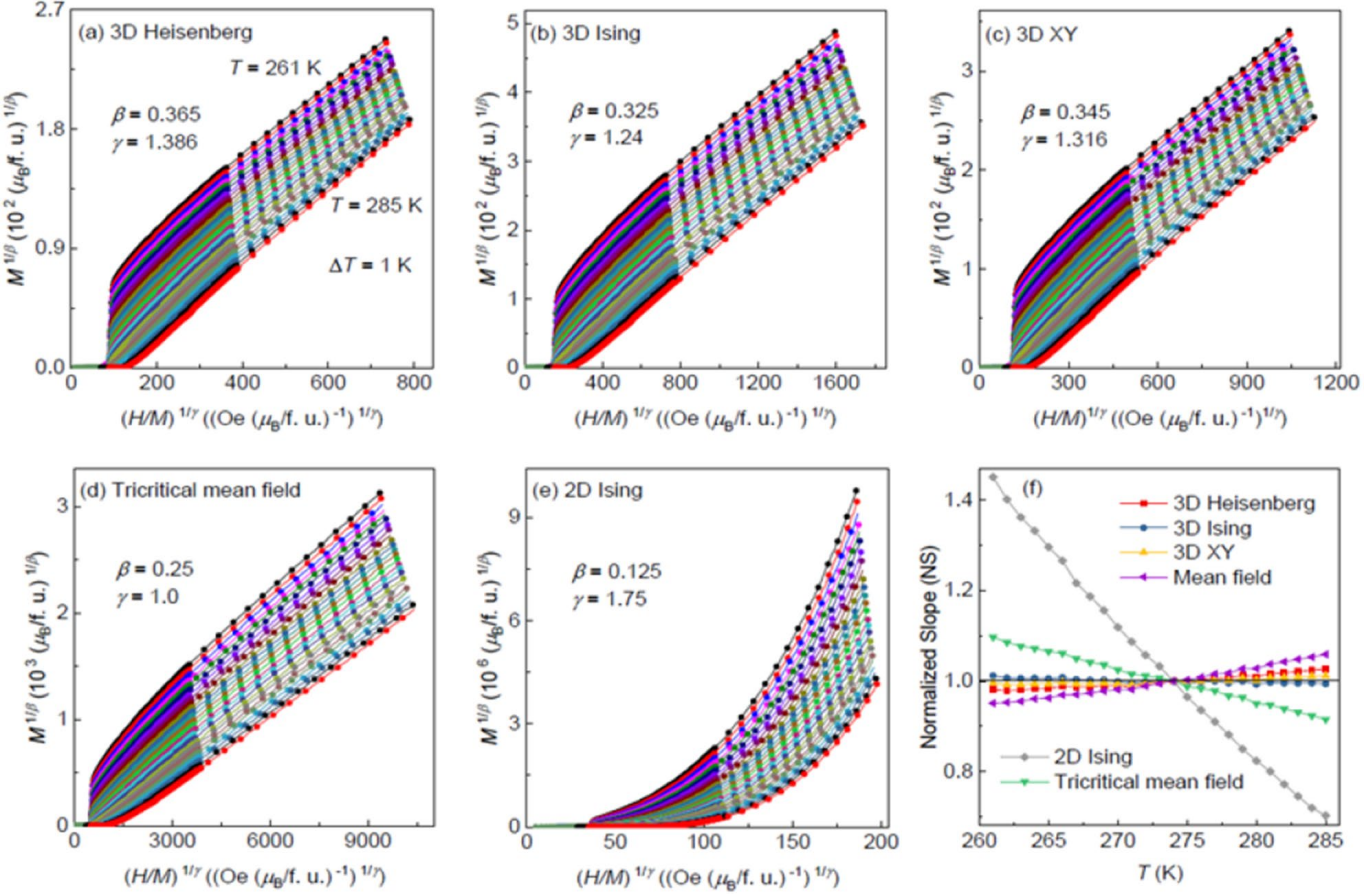
The isotherms of *M*^1/*β*^ versus (*H*/*M*)^1/*γ*^ with (**a**) 3D Heisenberg model, (**b**) 3D Ising model, (**c**) 3D XY model, (**d**) Tricritical mean-field model and (**e**) 2D Ising model. (**f**) Normalized slope versus temperature curves for six sets of critical exponents.

The second-order PM to FM phase transition in Fe_5_GeTe_2_ can be described by the magnetic equation of state and is characterized by critical exponents *β*, *γ* and *δ* that are mutually related. According to the scaling hypothesis, for a second-order phase transition, the spontaneous magnetization *M*_*S*_(*T*) below *T*_C_, the inverse initial susceptibility *χ*_0_^–1^(*T*) above *T*_C_ and the magnetization *M* at *T*_C_ can be used to obtain *β*, *γ* and *δ* by using the equations^36^:

1 $${M}_{S}\left(T\right)={M}_{0}{\left(-\varepsilon \right)}^{\beta },\varepsilon <0,T<{T}_{C},$$

2 $${\chi }_{0}^{-1}\left(T\right)=\left({h}_{0}/{m}_{0}\right){\varepsilon }^{\gamma },\varepsilon >0,T>{T}_{C},$$

3 $$\mathrm{and }\;\;M=D{H}^{1/\delta }, \varepsilon =0,T={T}_{C},$$

where *ε* = (*T*—*T*_C_)/*T*_C_ is the reduced temperature, and *M*_0_, *h*_0_/*m*_0_, and *D* are the critical amplitudes. Though the Landau mean-field theory can not be used, the critical isothermal magnetizations, alternatively, can be analyzed with the Arrott-Noakes equation of state^37^:

4 $${(H/M)}^{1/\gamma }=a\upvarepsilon +b{M}^{1/\beta },$$

where *a* and *b* are the fitting constants. Five different models including the 2D Ising model (*β* = 0.125, *γ* = 1.75)^38^, the 3D Heisenberg model (*β* = 0.365, *γ* = 1.386)^38^, the 3D Ising model (*β* = 0.325, *γ* = 1.24)^38^, the 3D XY model (*β* = 0.345, *γ* = 1.316)^39^ and the tricritical mean-field model (*β* = 0.25, *γ* = 1.0)^40^ were used for the modified Arrott plots, which are shown in Fig. 2a–e. One can see that the lines in Fig. 2d,e are not parallel to each other, thus excluding the tricritical mean-field and 2D Ising models. In Fig. 2a–c, all lines in each figure are almost parallel to each other in the high magnetic field region, thus making the choice of an appropriate model for Fe_5_GeTe_2_ impossible in this step. As we mentioned above, the modified Arrott plot should be a set of parallel lines in the high magnetic field region with the same slope of *S*(*T*) = *dM*^1*/β*^/*d*(*H/M*)^1/*γ*^. The normalized slope *NS* is defined by *NS* = *S*(*T*)/*S*(*T*_C_), which enables us an easy comparison of the *NS* of different models and to select out the most appropriate one with the ideal value of unity. The *NS* values versus the temperature for different models are plotted in Fig. 2f, which clearly show that the *NS* of the 2D Ising model has the largest deviation from unity. One can see that when *T* > *T*_C_*, NS* of the 3D Ising model is close to unity, while when *T* < *T*_C_ the 3D XY model seems as the best. This indicates that the critical behavior of Fe_5_GeTe_2_ may not belong to a single universality class. The fact also likely indicates that the magnetic character of Fe_5_GeTe_2_ is nearly isotropic above *T*_C_ and the enhancement of the anisotropic exchange below *T*_C_.

**Figure 3:**
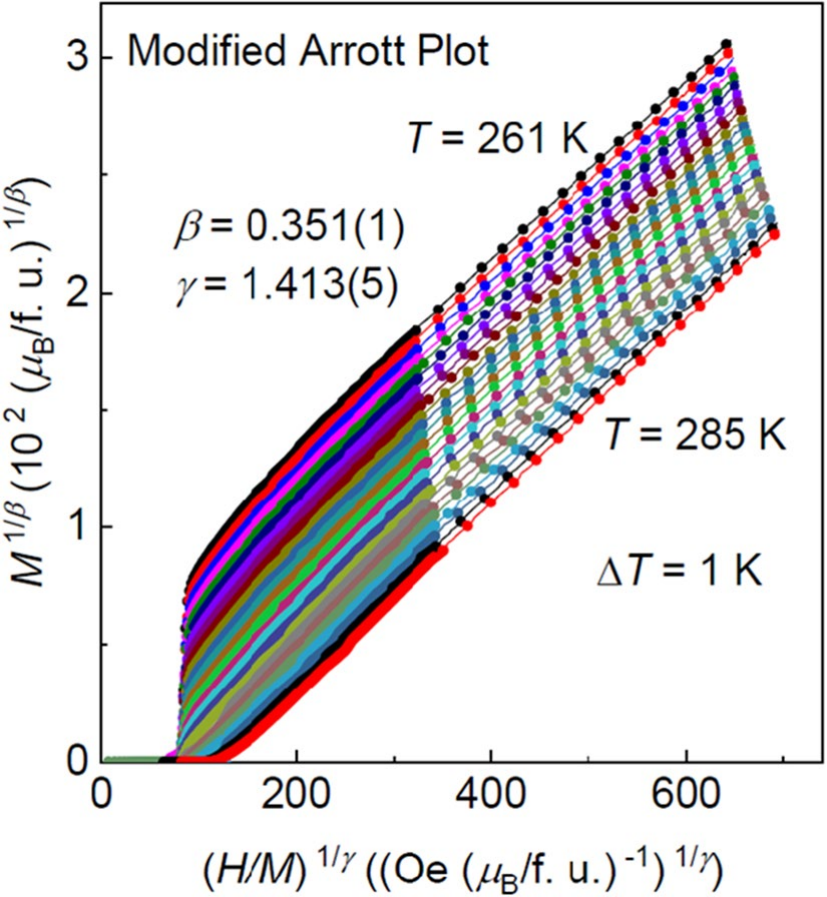
Modified Arrott plot of isotherms with *β* = 0.351(1) and *γ* = 1.413(5) for Fe_5_GeTe_2_.

**Figure 4:**
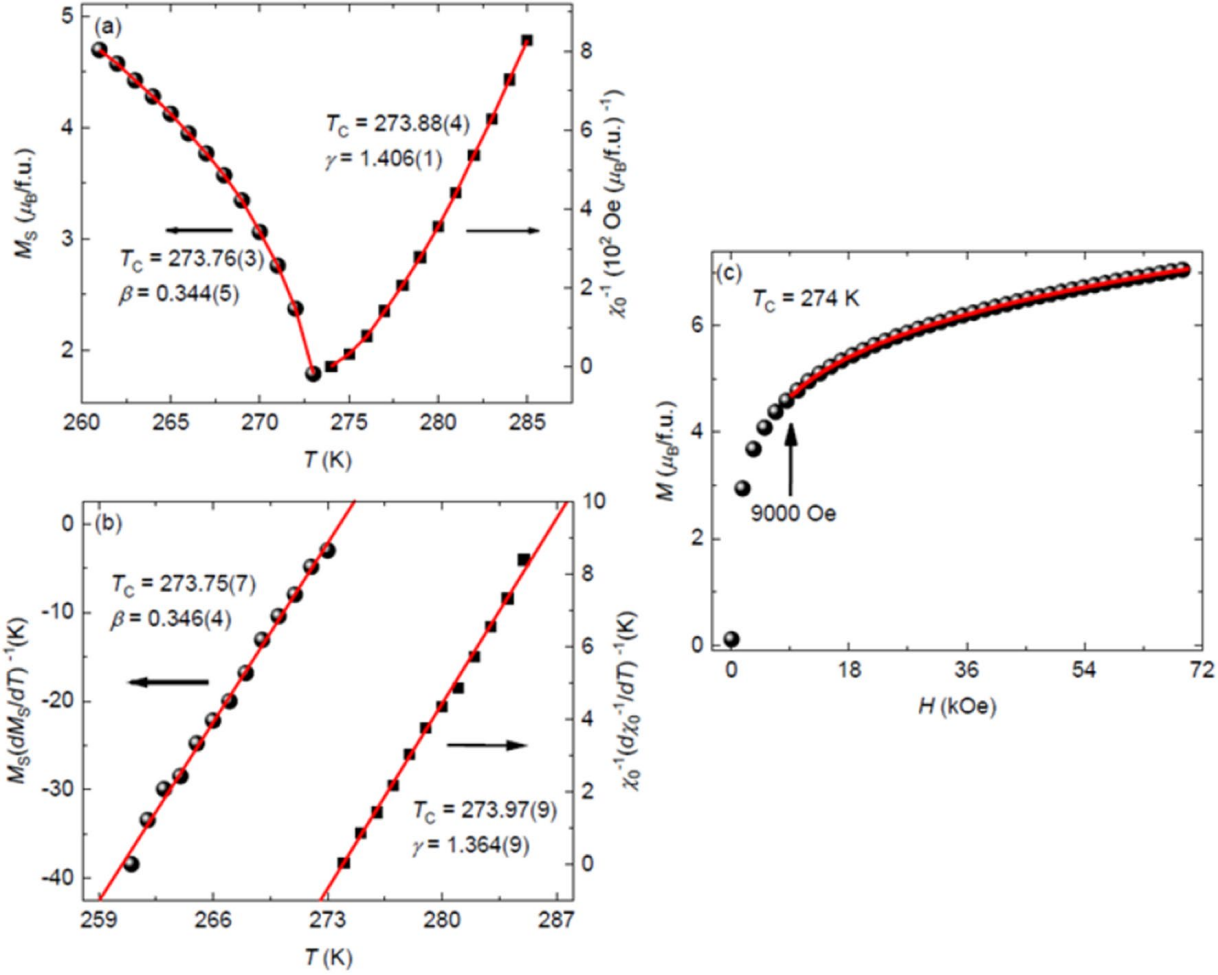
(**a**) Temperature dependence of the spontaneous magnetization *M*_S_ (left) and the inverse initial susceptibility ${\chi }_{0}^{-1}\left(T\right)$ (right) with solid fitting curves for Fe_5_GeTe_2_. (**b**) Kouvel-Fisher plots of *M*_*S*_(*T*)/(*dM*_*S*_(*T*)/*dT*) (left) and *χ*_0_^–1^(*T*)/(*dχ*_0_^–1^(*T*)/*dT*) (right) with solid fitting curves for Fe_5_GeTe_2_. (**c**) Isotherm *M*(*H*) collected at *T*_C_ = 274 K for Fe_5_GeTe_2_. Inset: the same plot in log–log scale with a solid fitting curve.

To achieve in-depth insights into the nature of the PM to FM transition in Fe_5_GeTe_2_, the precise critical exponents and critical temperature should be obtained. In the modified Arrott plot, the linear extrapolation of the nearly straight curves from the high magnetic field region intercepting the *M*^1/*β*^ and (*H*/*M*)^1/*γ*^ axes yields reliable values of *M*_*S*_(*T*) and *χ*_0_^–1^(*T*), respectively. The extracted *M*_*S*_(*T*) and *χ*_0_^–1^(*T*) can be used to fit the *β* and *γ* by using Eqs. (1) and (2). The thus obtained *β* and *γ* are thereafter used to reconstruct a modified Arrott plot. Consequently, new *M*_*S*_(*T*) and *χ*_0_^–1^(*T*) are generated from the linear extrapolation in the high field region, and a new set of *β* and *γ* will be acquired. This procedure should be repeated until *β* and *γ* are convergent. The obtained critical exponents from this method are independent on the initial parameters, thus guaranteeing the reliability of the analysis and that the obtained critical exponents are intrinsic. The final modified Arrott plot with *β* = 0.351(1) and *γ* = 1.413(5) is presented in Fig. 3, which shows that the isotherms in the high magnetic field region are actually a set of parallel straight lines. In addition, the final *M*_*S*_(*T*) and *χ*_0_^–1^(*T*) with solid fitting curves are depicted in Fig. 4a, which yield the critical exponents *β* = 0.344(5) with *T*_C_ = 273.76(3) K and *γ* = 1.406(1) with *T*_C_ = 273.88(4) K.

It is necessary to check the accuracy of above analysis. The Kouvel-Fisher (K-F) method can also be employed to fit the critical exponents and critical temperature, which is expressed as^40^:

5 $$\frac{{M}_{S}(T)}{d{M}_{S}(T)/dT}=\frac{T-{T}_{C}}{\beta }$$

6 $$\mathrm{and }\frac{{\chi }_{0}^{-1}\left(T\right)}{d{\chi }_{0}^{-1}\left(T\right)/dT}=\frac{T-{T}_{C}}{\gamma },$$

where *M*_*S*_(*T*)/(*dM*_*S*_(*T*)/*dT*) and *χ*_0_^–1^(*T*)/(*dχ*_0_^–1^(*T*)/*dT*) are linearly dependent on temperature with the slopes of 1/*β* and 1/*γ*, respectively. As is shown in Fig. 4b, the linear fits give *β* = 0.346(4) with *T*_C_ = 273.75(7) K and *γ* = 1.364(9) with *T*_C_ = 273.97(9) K, respectively, which are consistent with those obtained from the iterative modified Arrott plot, thus confirming the reliability of the above analysis.

The iterative modified Arrott plot gives the critical exponents *β* and *γ*, while the critical exponent *δ* can be obtained by using Eq. (3). Figure 4c shows the isothermal magnetization *M*(*H*) at a critical temperature *T*_C_ = 274 K and the inset shows the plot at a log–log scale. According to Eq. (3), the *M*(*H*) at *T*_C_ should be a straight line in the log–log scale with the slope of 1/*δ*, thus giving *δ* = 5.02(1). To check the reliability of such analysis, *δ* was also calculated by using the Widom scaling relation^41^:

7 $$\delta =1+\frac{\gamma }{\beta },$$

which gives *δ* = 5.02(6) and *δ* = 4.94(0) by using the *β* and *γ* obtained with modified Arrott plot and Kouvel-Fisher plot, respectively, which are consistent with those fitted by using Eq. (3).

**Figure 5:**
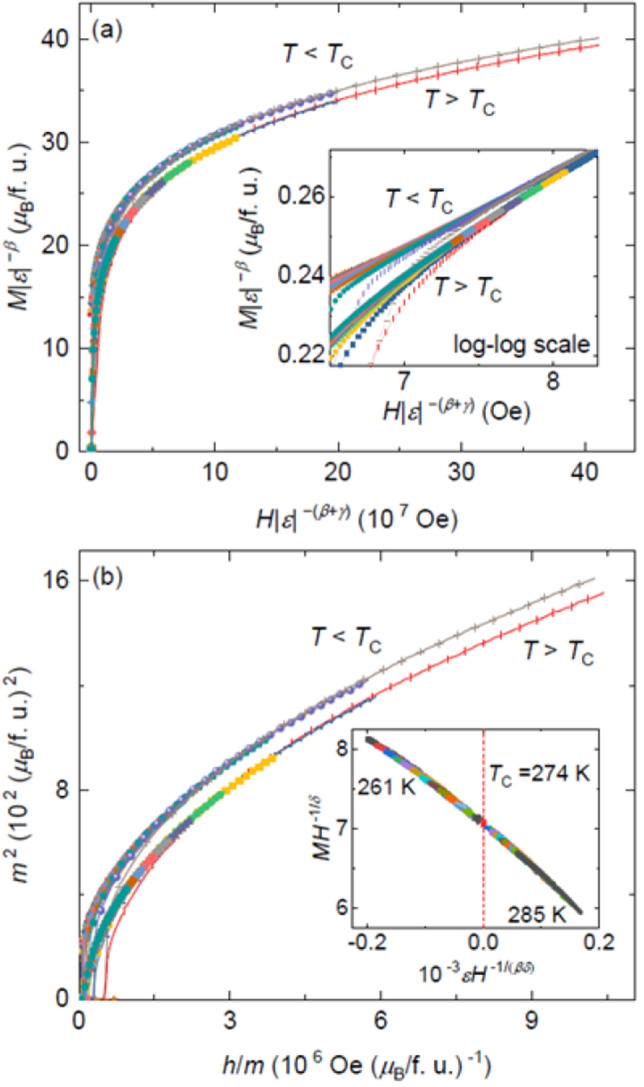
(**a**) The $m\equiv {\varepsilon }^{-\beta }M(H,\varepsilon )$ as a function of the $h\equiv {\varepsilon }^{-(\beta +\gamma )}$ below and above *T*_C_ for Fe_5_GeTe_2_. Inset is the same *m*(*h*) data in log–log scale. (**b**) Plot in the form of *m*^2^(*h*/*m)* for Fe_5_GeTe_2_. Inset shows the plot of *εH*^−(*βδ*)^ vs. *MH*^−1/*δ*^ below and above *T*_C_.

From above analysis, a set of critical exponents are obtained, which are actually self consistent. It is of essential importance to check whether the obtained critical exponents and *T*_C_ can generate a scaling equation of state for Fe_5_GeTe_2_, i.e., to examine the reliability of these critical exponents again by using the scaling analysis. According to the scaling hypothesis, for a magnetic system in the critical asymptotic region, the scaling equation of state can be expressed as^42^:

8 $$M\left(H,\varepsilon \right)={\varepsilon }^{\beta }{f}_{\pm }(\frac{H}{{\varepsilon }^{\beta +\gamma }})$$

where *M*(*H*, *ε*), *H*, and *T* are variables; *f*_+_ for *T* > *T*_C_ and *f*_˗_ for *T* < *T*_C_ are the regular functions. Equation (8) can also be written as:

9 $$m={f}_{\pm }(h),$$

where $m\equiv {\varepsilon }^{-\beta }M(H,\varepsilon )$ and $h\equiv {\varepsilon }^{-(\beta +\gamma )}$. If the critical exponents *β*, *γ* and *δ* could be properly chosen, the scaled *m*(*h*) plot will fall onto two universal curves for *T* > *T*_C_ and *T* < *T*_C_, respectively. In such case, the interactions are believed to be properly renormalized in the critical regime following the scaling equation of state. The scaled *m* and *h* curves are plotted in Fig. 5a, which actually show two branches below and above *T*_C_, thus guarantying the reliability of the obtained critical exponents. The two branches are much clear when the same data are plotted in a log–log form, seen by the inset of Fig. 5a. To support the analysis, we used a more rigorous method by plotting *m*^2^ against *h*/*m*, seen in Fig. 5b in which all data apparently separate into two curves below and above *T*_C_. The reliability of the obtained critical exponents and *T*_C_ can also be examined by checking the scaling of the magnetization curves. The scaling state equation of magnetic systems is^42^:

10 $$\frac{H}{{M}^{\delta }}=h \left(\frac{\varepsilon }{{H}^{1/\beta }}\right),$$

where *h*(*x*) is a scaling function. From Eq. (10), the *εH*^−(*βδ*)^ vs. *MH*^−1/*δ*^should fall on one universal curve^43^, as seen by the inset of Fig. 5b. The *T*_C_ lies on the zero point of *εH*^-(*βδ*)^ axis. As a result, the well rescaled curves further confirm that the obtained critical exponents and *T*_C_ are reliable and consistent with the scaling hypothesis.

It is valuable to compare the critical exponents of Fe_5_GeTe_2_ with those of other layered vdW magnets and those predicted by various models. The critical exponents of Fe_5_GeTe_2_ obtained by using different analysis techniques and different theoretical models are summarized in Table 1, together with those of other several FM vdW magnets including Fe_3-*x*_GeTe_2_ (*x* = 0, 0.15, and 0.36), Cr_2_Si_2_Te_6_, and Cr_2_Ge_2_Te_6_. The previous comprehensive study reached a conclusion that the critical exponent *β* for a 2D magnets lies in the range of ~ 0.1 ≤ *β* ≤ 0.25^44^. It is apparent that the *β* values of Cr_2_Si_2_Te_6_ and Cr_2_Ge_2_Te_6_, which were verified as 2D Ising magnets^45,46^, are actually within the window, while those of Fe_3-*x*_GeTe_2_ and Fe_5_GeTe_2_ are apparently larger than 0.25, thus excluding the 2D Ising model for them^29,30^. Moreover, the *γ* values of Fe_3-*x*_GeTe_2_ and Fe_5_GeTe_2_ are much larger than those for the tricritical mean-field and 3D Ising models^38,39^, suggesting the two models are not appropriate. Combining the *β* and *γ* values, the magnetic critical behavior in Fe_5_GeTe_2_ should have a 3D nature, indicating that the interlayer magnetic exchange can not be neglected. It was suggested that Fe_3-*x*_GeTe_2_ has a smaller vdW gap and hence a stronger interlayer magnetic exchange than that in Cr_2_(Si,Ge)_2_Te_6_^17^. It is therefore a natural hypothesis that the vdW gap in Fe_5_GeTe_2_ is also very small. To achieve more insights, the critical exponents of Fe_5_GeTe_2_ should be compared with the several 3D models more carefully. The *β* of Fe_5_GeTe_2_ is much closer to that of the 3D XY model^39^ while the *γ* is closer to that of the 3D Heisenberg model^38^, likely implying that the obtained critical exponents of Fe_5_GeTe_2_ can not be simply categorized into any conventional universality classes.

**Table 1:** A summary of the critical exponents of Fe_5_GeTe_2_, Fe_3-x_GeTe_2_, Cr_2_Si_2_Te_6_, Cr_2_Ge_2_Te_6_ and those predicted by different models (MAP: Modified Arrott plot; KF: Kouvel-Fisher method; CI: critical isotherm analysis).

Composition	References	Technique	*β*	*γ*	*δ*	{*d*:*n*}	*J*(*r*)
Fe_5_GeTe_2_	This work	MAP	0.351 (1)	1.413 (5)	5.02 (6)	{3:3}	*r*^–4.916^
	This work	KF	0.346 (4)	1.364 (9)	4.94 (0)		
	This work	CI			5.02 (1)		
3D Heisenberg	^4^	Theory	0.365	1.386	4.8		
3D XY	^4^	Theory	0.345	1.316	4.81		
3D Ising	^4^	Theory	0.325	1.24	4.82		
Tricritical mean field	^5^	Theory	0.25	1.0	5		
Mean field	^4^	Theory	0.5	1.0	3		
Fe_2*.*64_Ge_0*.*87_Te_2_	^12^	KF	0.372 (4)	1.265 (1)	4.401 (6)	{3:3}	*r*^–4.89^
Fe_2*.*85_GeTe_2_	^13^	KF	0.363	1.228	4.398		*r*^–4.8^
Fe_3_GeTe_2_	^3^	KF	0.322 (4)	1.063 (8)	4.301 (6)		*r*^–4.6^
Cr_2_Si_2_Te_6_	^14^	KF	0.175 (9)	1.562 (9)	9.925 (5)	{2:1}	*r*^–3.63^
Cr_2_Ge_2_Te_6_	^15^	KF	0.200 (3)	1.28 (3)	7.405	{2:1}	*r*^–3.52^

For a homogenous magnet, it is essential to use the magnetic exchange distance *J*(*r*) to further determine the universality class of the magnetic phase transition. Within the framework of to the renormalization group theory, the magnetic exchange decays with the distance *r* in a form *J*(*r*) ~ e^–*r*/*b*^ for the short-range magnetic exchange and *J*(*r*) ~ *r*^–(*d*+*σ*)^ for the long-range exchange, where *r* is the exchange distance, *b* is the spatial scaling factor, *d* is the dimensionality of the system, and the positive constant *σ* denotes the range of exchange interaction^47,48^. Moreover, within this theory model the magnetic susceptibility exponent *γ* is defined as^47^:

11 $$\gamma =1+\frac{4}{d}\left(\frac{n+2}{n+8}\right)\Delta \sigma +\frac{8(n+2)(n-4)}{{d}^{2}{(n+8)}^{2}}\left[1+\frac{2G(\frac{d}{2})(7n+20)}{(n-4)(n+8)}\right]{\Delta \sigma }^{2},$$

where *n* is the spin dimensionality, Δ*σ* = (*σ* – *d*/2) and $G\left(\frac{d}{2}\right)=3-\frac{1}{4}{(\frac{d}{2})}^{2}$. For 3D materials (*d* = 3) with 3/2 ≤ *σ* ≤ 2, the magnetic exchange decays relatively slowly as *J*(*r*) ~ *r *^–(*d*+*σ*)^ due to a long-range magnetic exchange. For *σ* > 2, the 3D Heisenberg model is valid for 3D isotropic magnets, where *J*(*r*) decreases faster than *r* ^-5^ due to the short-range magnetic exchange, while when *σ* ≤ 3/2, the mean-field model works and *J*(*r*) decreases slower than *r*^-4.5^^47,48^. To obtain the values of *d*, *n*, and *σ* for Fe_5_GeTe_2_, a method similar to that in Ref.^47^. was adopted. In this method, *σ* is initially adjusted according to Eq. (11) with several sets of {*d* : *n*} to get a proper *γ* that is close to the experimental value (~ 1.364). The obtained *σ* is then used to calculate other critical exponents by the following equations: *ν* = *γ*/*σ*, *α* = 2 − *νd*, *β* = (2 − *α* − *γ*), and *δ* = 1 + *γ*/*β*. Several sets of {*d* : *n*} will be tried, with the typical results being summarized Table 2, which finally achieved the critical exponents of *β* = 0.3851, *γ* = 1.3613 and *δ* = 4.5351, which match well with the experimental values, when {*d*: *n*} = {3: 3} and *σ* = 1.916. Such a result indicates that the 3D Heisenberg type magnetic exchange with long-range interaction decaying as *J*(*r*) ≈ *r*^–4.916^ can account for the magnetic properties of Fe_5_GeTe_2_, which is consistent with our analysis presented above.

**Table 2:** Critical exponents calculated by the renormalization group theory.

*d*	*n*	*σ*	*β*	*γ*	*δ*
3	3	1.9160	0.3851	1.3613	4.5351
2	1	1.3603	0.3168	1.370	5.3241
2	3	1.2740	0.3904	1.370	4.5096

The magnetic exchange in quasi-2D vdW magnets has been subjected to immense investigations. For Cr_2_Si_2_Te_6_, the magnetic critical behavior analysis and neutron scattering studies consistently suggest the universality class of 2D Ising model accounting for its magnetic properties^26,49^. Because of the smaller vdW gap and hence an enhanced interlayer exchange in Cr_2_Ge_2_Te_6_, its critical behavior shows a transition from the 2D Ising-type to a 3D tricritical mean-field type^50^. It is useful to compare the magnetic critical behavior of Fe_5_GeTe_2_ with that of Fe_3_GeTe_2_. The mean distance between the two adjacent Te layers that across the vdW gap in Fe_3_GeTe_2_ is 0.423 nm^51^, which is rather close to that of CrGeTe_3_, 0.377 nm^49^, which presumably can account for the 3D Heisenberg characteristics of the critical behavior. Previous studies on Fe_5_GeTe_2_ indicate small magnetic anisotropy at high temperature^20^, so the 3D magnetism for the critical behavior in Fe_5_GeTe_2_ is reasonable. Moreover, it is found that the magnetic anisotropy in Fe_3-*x*_GeTe_2_ strongly depends on the Fe deficiency^52^, which can be largely suppressed with increasing the deficiency content *x*. If we pay a close attention to the critical exponents of Fe_5_GeTe_2_, it is easily found that they are much closer to those of Fe deficient Fe_3-*x*_GeTe_2_^29^, likely further demonstrating the weak magnetic anisotropy in Fe_5_GeTe_2_. However, the possible transition between different universality classes of models of the critical behavior should be carefully checked, if we recall into our mind that a critical phase transition between 3 and 2D at the temperature of ~ 0.9*T*_C_ in NiPS_3_ and an anisotropic 2D to 3D magnetism below *T*_C_ in MnPS_3_ were experimentally confirmed^53,54^. Though such possibility has not been examined yet in Fe_3-*x*_GeTe_2_, considering that Fe_3-*x*_GeTe_2_ indeed shares similarities as *M*PS_3_ (*M* = Mn, Fe, and Ni) in that they all have 2D antiferromagnetic ground state with the ferromagnetic layers in them order antiferromagnetically along the *c*-axis at low temperature, as well as the 3D critical behavior near *T*_C_, the critical phase transition definitely need to be checked in Fe_3-*x*_GeTe_2_. For Fe_5_GeTe_2_, it is somewhat different from *M*PS_3_ and Fe_3-*x*_GeTe_2_, which behaves as an easy-axis vdW ferromagnet with the magnetic moments preferring to align along the *c*-axis but with weak anisotropy at high temperature due to the easy polarization of moments and the interaction between the FM layers is still FM. However, the magnetism of Fe_5_GeTe_2_ is somewhat complex due to the multiple Fe sublattices and composition tunable *T*_C_. It is revealed that the magnetic moments on Fe(1) sublattice order below ~ 100–120 K while the majority of the moments order at *T*_C_^21^. Short-range order associated with occupations of split sites of Fe(1) is also present. Additionally, the magnetic anisotropy is enhanced at low temperature. Regarding these, more studies to establish the precise spin structure at low temperature are extremely desired.

## Conclusion

In summary, we have investigated the magnetic critical behavior in vicinity of the PM to FM phase transition in the quasi-2D van der Waals ferromagnet Fe_5_GeTe_2_ which has a near room temperature *T*_C_ of approximately 270 K. The estimated critical exponents *β, γ* and *δ* values from the various techniques and theoretical models show nice consistence with each other and follow the scaling behavior well. The critical exponents suggest a second order phase transition and they do not belong to any single universality class of model, just lying between the 3D Heisenberg model and the 3D XY model. The magnetic exchange distance is found to decay as *J*(*r*) ≈ *r*^–4.916^, which is close to that of 3D Heisenberg model with long-range exchange. The critical phenomena indicate weak magnetic anisotropy of Fe_5_GeTe_2_ at high temperature, possibly due to its small vdW gap. The very recent calculations indicate that monolayer formation energy of Fe_5_GeTe_2_ lies inside the energy range of other 2D materials^55^, and the synthesis of the monolayer is therefore highly expected. Moreover, considering the tunable *T*_C_ which can even to be ~ 350 K^20,21,56^, the investigation on the precise magnetic structure of Fe_5_GeTe_2_ would find extraordinary opportunities for applications in next-generation spintronic devices.

## Methods

Single crystals were grown from chemical vapor transport (CVT) technique by using iodine as the transport agent, similar as the method described previously^20,21^. The crystal used in this experiment is flat with a typical dimension of 2 mm * 2 mm * 0.1 mm. The crystallographic phase and crystal quality were examined on a Bruker D8 single crystal X-ray diffractometer (SXRD) with Mo *K*_α_ (λ = 0.71073 Å) at 300 K. The chemical compositions and uniformity of stoichiometry were checked by the energy dispersive spectroscopy (EDS) at several spots on the crystals. The direct current (dc) magnetization was measured on the Quantum Design magnetic properties measurement system (MPMS-3) with the magnetic field applied parallel to *c*-axis of the crystal. Isothermal magnetizations were collected at a temperature interval of 1 K in the temperature range of 261–285 K, which is just around *T*_C_ (~ 270 K). It should be noted that each curve was initially magnetized. The applied magnetic field was corrected by considering the demagnetization factor, which was used for the analysis of critical behavior. The demagnetization factor is roughly estimated to be ~ 0.88 with considering the crystal size^57^.

## Supplementary information

Supplementary Information 1.
