# Supplementary material for: Magnetic critical behavior of the van der Waals Fe5GeTe2 crystal with near room temperature ferromagnetism
Source: Sci Rep. 2020 Sep 18;10:15345. doi: 10.1038/s41598-020-72203-3 (PMC7501290; doi:10.1038/s41598-020-72203-3)
Supplement: Supplementary file 1 — Supplementary Information 1. [file 41598_2020_72203_MOESM1_ESM.docx]

**Supplemental materials**

**Magnetic critical behavior of the van der Waals Fe_5_GeTe_2_ crystal with near room temperature ferromagnetism**

Zhengxian Li^1,2,3^, Wei Xia^1,2,3^, Hao Su^1,2,3^, Zhenhai Yu^1^, Yunpeng Fu^1^, Leiming Chen^4*^, Xia Wang^1,5^, Na Yu^1,5^, Zhiqiang Zou^1,5^, Yanfeng Guo^1^*^*^*

*^1^School of Physical Science and Technology, ShanghaiTech University, Shanghai 201210, China*

*^2^Shanghai Institute of Optics and Fine Mechanics, Chinese Academy of Sciences, Shanghai 201800, China*

*^3^University of Chinese Academy of Sciences, Beijing 100049, China*

*^4^School of materials science and engineering, Henan key laboratory of aeronautic materials and application technology, Zhengzhou University of Aeronautics, Zhengzhou, Henan, 450046*

*^5^Analytical Instrumentation Center, School of Physical Science and Technology, ShanghaiTech University, Shanghai 201210, China*

^*^E-mails:

[lmchen@zua.edu.cn](mailto:lmchen@zua.edu.cn),

[guoyf@shanghaitech.edu.cn](mailto:**guoyf@shanghaitech.edu.cn).

1. **

Phase and quality chracterizations on Fe_5_GeTe_2_ crystal**

Fig. S1. (a-b) Crystal structure of Fe_5_GeTe_2_ viewed from directions along the *a*- and *c*-axes, respectively. (c) Image of a typical Fe_5_GeTe_2_ single crystal. (d-f) Single crystal X-ray diffraction patterns in the reciprocal space along the (*0 k l*), (*h 0 l*), and (*h k 0*) directions.

The SXRD measurement gives the space group $R\bar{3}m$ (No.166) of the crystal structure with lattice parameters *a* = 4.05(1) Å, *c* = 29.23(2) Å, and *α* = *β* = 90°, *γ* = 120°, consistent with previously reported values in Ref. 2 but slightly larger than those in Ref. 1, presumably due to the smaller Fe deficiency content in our crystals, which is determined as *x* ~ 0.09 in Fe_5-_*_x_*GeTe_2_ by the energy dispersive spectroscopy (EDS) chracterizations, which could be seen below. We thereafter still use Fe_5_GeTe_2_ to represent the used crystals in this experiment. The crystal structure, schematically shown in Figs. S1(a)-(b) seen from the *a*- and *c*-axis respectively, is drawn based on analyzing the SXRD data. The structure of Fe_5_GeTe_2_, analogue to Fe_3_GeTe_2_, is basically built up by 2D slabs of Fe and Ge between the van der Waals gapped Te layers.^2, 3^ The optical image of the typical crystal is shown in Fig. S1c. The perfect reciprocal space lattice of SXRD without any other miscellaneous points, seen in Figs. S1(d)-(f), indicates pure phase and high quality of the crystal. Previous studies unveiled that Fe_5_GeTe_2_ has two similar crystal structures when the synthesis methods are different.^3^ One structure has a higher symmetry in a space group $R\bar{3}m$ and the other one has the space group of *R3m*. These structures both are with rhombohedral lattice centering three Fe_5_GeTe_2_ layers in each unit cell. The structure with a higher symmetry contains three Fe sites in each unit cell, where the Fe(1) site, marked in Fig. S1(a), is treated as a split site occupying either above or below the neighboring Ge site. As a contrast, in the lower symmetry model the equivalent Fe(1) site is never treated as a split site and is always “up” in a layer. Analogue to Fe_3-_*_x_*GeTe_2_, the equivalent Fe(1) site could be vacant, thus allowing the Fe deficiency in both compounds to tune the *T*_C_.

1. **The EDS measurements on Fe_5_GeTe_2_ crystal**


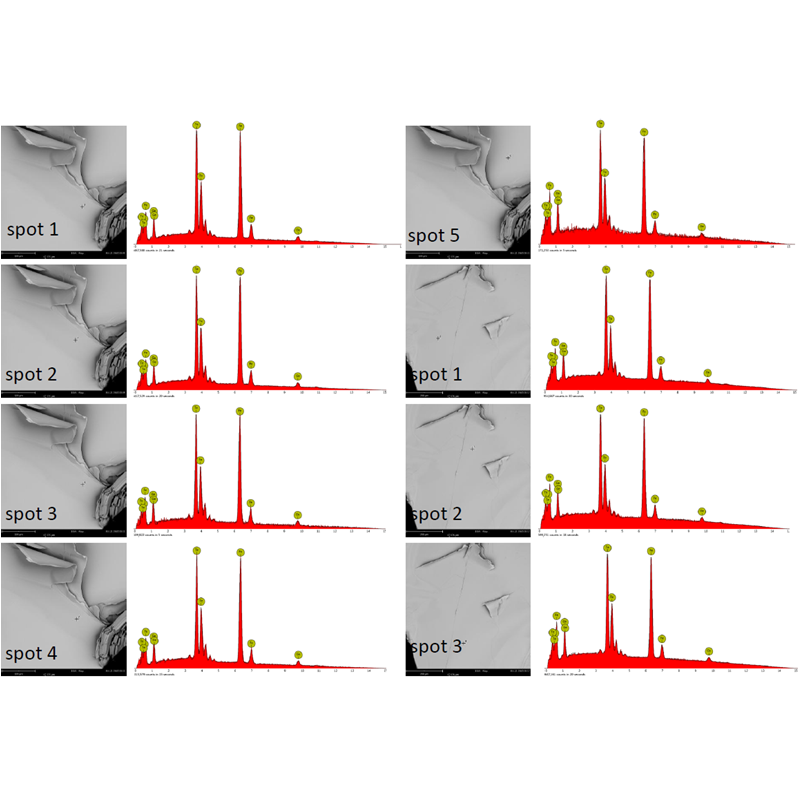
The Element analysis of Fe_5_GeTe_2_ was performed by using energy-dispersive X-ray spectroscopy (EDS). More than 3 random areas on the surface of the crystal were measured, as shown by several typical pictures shown in Figure S2, and more than 10 spots in each area were measured. The values were finally averaged, which are presented in Table S1, showing the compositions as Fe : Ge : Te = 4.91 : 1.01 : 2.08.

Fig. S2. Several typical EDS measurement results of the Fe_5_GeTe_2_ single crystal.

Table. S1. The averaged values of the EDS results of the Fe_5_GeTe_2_ single crystal.

**Reference**

1. A. F. May, C. A. Bridges, and M. A. McGuire, Phys. Rev. Mater. **3**, 104401 (2019).
2. May, A. F. *et al*. [Ferromagnetism Near Room Temperature in the Cleavable van der Waals Crystal Fe_5_GeTe_2_](https://pubs.acs.org/doi/10.1021/acsnano.8b09660). ACS Nano **13**, 4436-4442 (2019).
3. Stahl, J., Shlaen, E., & Johrendt, D. The van der Waals Ferromagnets Fe_5–δ_GeTe_2_ and Fe_5–δ–_*_x_*Ni*_x_*GeTe_2_ - Crystal Structure, Stacking Faults, and Magnetic Properties. Z. Anorg. Allg. Chem. **644**, 1923-1929 (2018).
